# Bacterial diseases in marine fish species: current trends and future prospects in disease management

**DOI:** 10.1007/s11274-023-03755-5

**Published:** 2023-09-25

**Authors:** Avani Hegde, Suhani Kabra, Renuka Manjunath Basawa, Dnyanada Anil Khile, Rahil Ummar Faruk Abbu, Naomi Ann Thomas, Nava Bharati Manickam, Ritu Raval

**Affiliations:** 1 Department of Biotechnology, Manipal Institute of Technology (MIT), Manipal Academy of Higher Education (MAHE), Manipal, Karnataka 576104 India; 2 Manipal Biomachines, Manipal Institute of Technology (MIT), Manipal Academy of Higher Education (MAHE), Manipal, Karnataka 576104 India

**Keywords:** Economy, Disease, Bacterial fish pathogens, Antibiotics, Vaccination, Antimicrobials, Probiotics

## Abstract

The fisheries sub-sector of aquaculture—i.e., the pisciculture industry, contributes significantly to a country’s economy, employing a sizable proportion of the population. It also makes important contributions to household food security because the current demand for animal protein cannot be fulfilled by harvesting wild fish from riverines, lakes, dams, and oceans. For good pond management techniques and sustaining fish health, the fisherfolk, and the industry require well-established regulatory structures, efficient disease management strategies, and other extended services. In rearing marine fish, infections resulting from disease outbreaks are a weighty concern because they can cause considerable economic loss due to morbidity and mortality. Consequently, to find effective solutions for the prevention and control of the major diseases limiting fish production in aquaculture, multidisciplinary studies on the traits of potential fish pathogens, the biology of the fish as hosts, and an adequate understanding of the global environmental factors are fundamental. This review highlights the various bacterial diseases and their causative pathogens prevalent in the pisciculture industry and the current solutions while emphasising marine fish species. Given that preexisting methods are known to have several disadvantages, other sustainable alternatives like antimicrobial peptides, synthetic peptides, probiotics, and medicinal treatments have emerged to be an enormous potential solution to these challenges.

**Graphical abstract:**

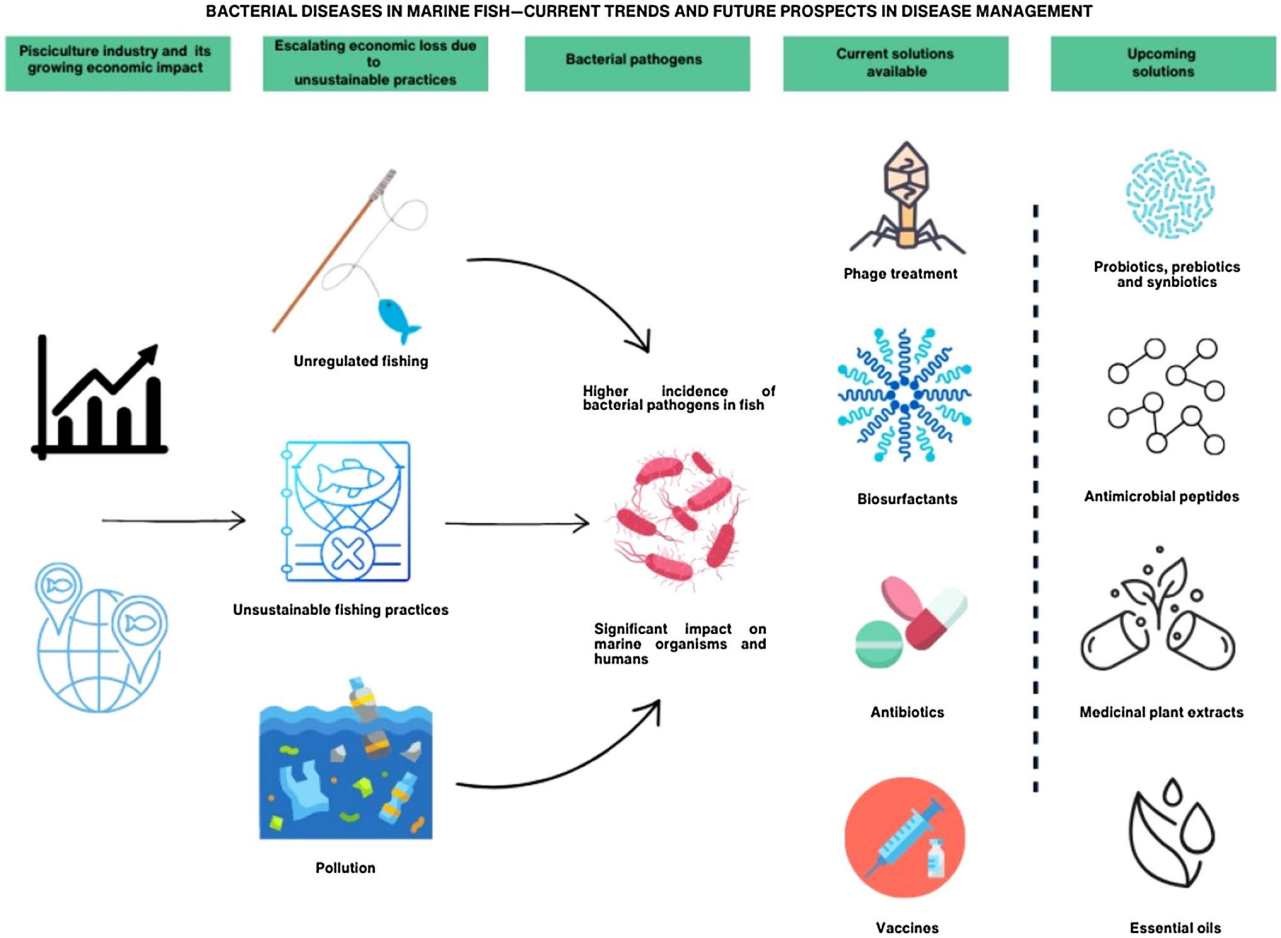

## Introduction

The production of seafood has been transformed by the aquaculture sector, which is one of the world’s fastest-growing food production technologies in recent decades. As a result, seafood is now a more significant food source on a global scale. The world’s population is expected to reach 10 billion by 2050, and to meet the growing and increasingly affluent population’s dietary needs, food production needs to expand by up to 56 per cent globally (“The State of World Fisheries and Aquaculture 2022,” 2022). In response to the increasing demand for marine protein, marine aquaculture offers a chance to boost seafood production.

Mariculture, or the farming of marine species, is an emerging industry that has drawn a desire for its potential to grow and diversify food systems, with approximately 249 different species farmed (Gentry et al. 2023). It is primarily known to be a major sub-sector of the aquaculture industry by offering possibilities for sustainable food production and the local communities’ economic development.

Commercial mariculture production is currently active in 102 different countries and on all the continents except Antarctica. The most recent annual production of more than 30 million metric tonnes reflects a nearly five-fold growth in mariculture productivity throughout the past 30 years (Gentry et al. 2023). However, it is primarily concentrated in fewer countries, with China exclusively accounting for more than one-third of the world’s total production (Gentry et al. 2023). The global food supply, in addition to per-capita consumption of fish and its associated products, continues to increase more rapidly than the world’s population. Thus, aquaculture production in marine waters expanded at a compound annual rate of 5.2 per cent from 2000 to 2018, while the whole aquaculture industry had yearly growth of 5.6 per cent (World Aquaculture 2022—A Brief Overview—Bartley, D.M.—Google Books). In 2020, 178 million tonnes of aquatic animals were projected to be produced worldwide, out of which 63 per cent (112 million tonnes) of the total production was cultivated in marine waters with 70 per cent through capture fisheries and 30 per cent from aquaculture (“The State of World Fisheries and Aquaculture 2022,” 2022).

**Fig. 1:**
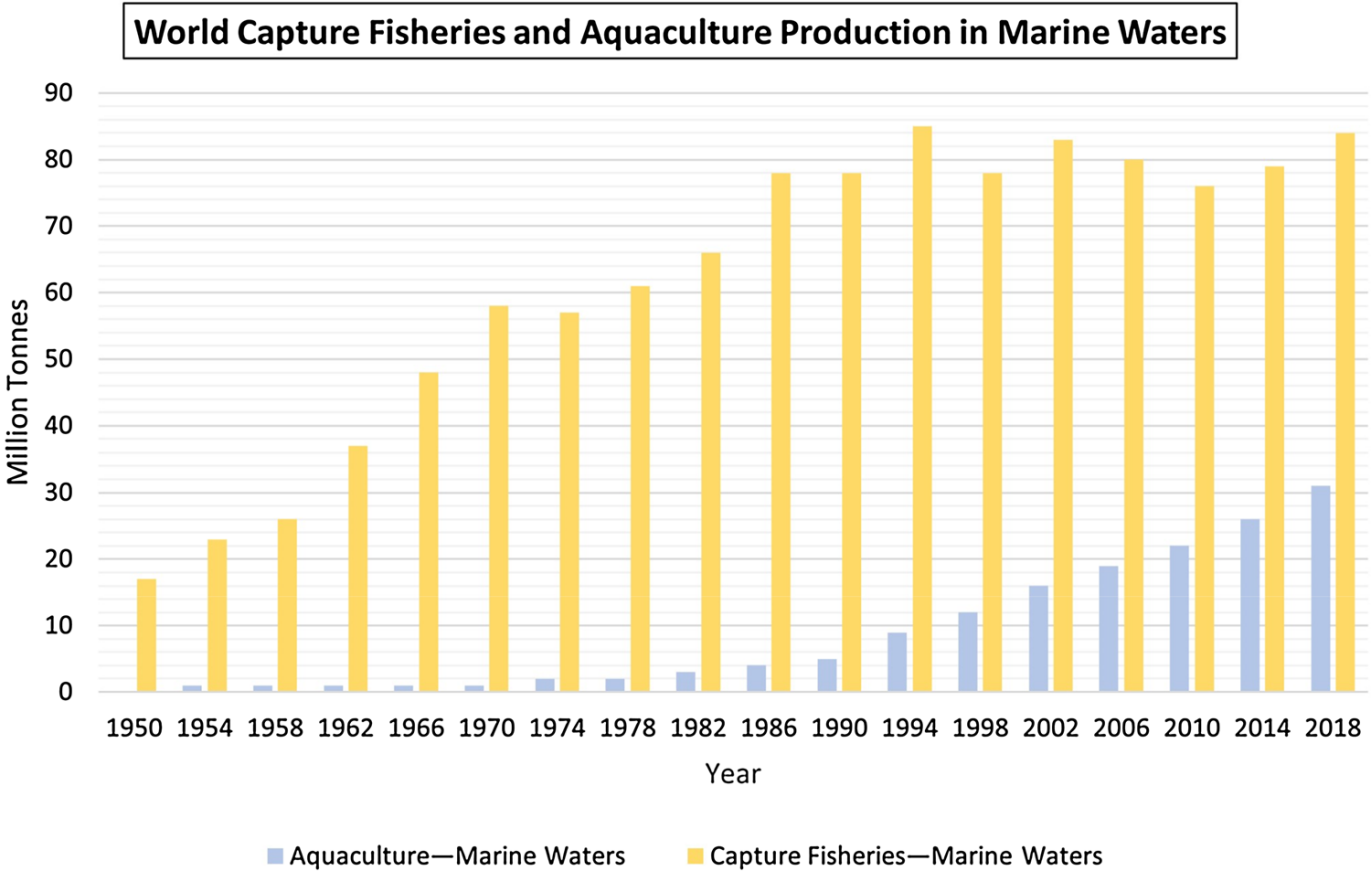
A bar graph indicating the overall increasing trend of world capture fisheries and aquaculture production spanning about seven decades between 1950 and 2018. (“The State of World Fisheries and Aquaculture 2020,” 2020)

Figure 1 demonstrates that the number of fish produced from marine areas increased to 84.4 million tonnes in 2018 from 81.2 million in 2017, with marine capture fisheries contributing to much of the growth (“The State of World Fisheries and Aquaculture 2022,” 2022). For numerous fish species, capture fisheries in marine waters continue to be the primary production source (they are projected to account for 44% of all aquatic animal produce in 2020). After several decades of progressive expansion, marine capture fisheries have remained consistent since the late 1980s at around 80 million tonnes, and in 2020, global marine captures were 78.8 million tonnes (“The State of World Fisheries and Aquaculture 2022,” 2022).

In 2020, a projected 58.5 million people were employed in fisheries and aquaculture. Aquaculture accounted for 35 per cent of employment, while capture fisheries accounted for 65 per cent (“The State of World Fisheries and Aquaculture 2022,” 2022). Albeit the mariculture industry provides sustenence to various communities, it still encounters significant obstacles that, in some situations, make it intricate to provide sustainable results (Naylor et al. 2021).

Despite the considerable influence the industry has had on food supply, various environmental and health variables can influence and cause illnesses in marine fish, leading to huge financial losses. These illnesses include those brought on by pathogenic bacteria, more especially by the Gram-negative bacterial genera and, to a lesser extent, the Gram-positive bacterial genera (Maldonado-Miranda et al. 2022). Due to the enormous number of species raised in various aquaculture systems, research on novel illnesses and the range of susceptible host species frequently lags advancements in aquaculture. Furthermore, those who are accountable for preserving biosecurity frequently take a slow collective awareness of emerging dangers (“The State of World Fisheries and Aquaculture 2020,” 2020). There is frequently a paucity of fundamental information regarding the pathogen—pathogenicity and transmission channels—and its host(s)—species, life stages infected, immunity, and genetics. Several bacterial infections in fish species, including *Aeromonas salmonicida*, *Pseudomonas anguilliseptica*, *Vibrio harveyi* and *V. anguillarum*, *Moritella viscosa*, *Tenacibaculosis*, and *Lactococcus garvieae*, have profoundly affected a variety of economically important fish species reared in marine and brackish water aquaculture production around the world causing heavy financial losses for the aquaculture industry worldwide (Irshath et al. 2023). This paper outlines the common bacterial infections in marine fish and highlights the need for more sustainable solutions by elucidating on the current and upcoming solutions, both in pre-clinical and commercial stages of use.

## Major bacterial diseases in marine fish

Disease occurrences are now a ruling obstacle to the production and trade of sustainable aquaculture products, impacting fisherfolk’s socioeconomic standing in developing nations worldwide. Outbreaks by opportunistic pathogens can be brought on by a multitude of stress conditions such as the inadequate physicochemical and microbiological quality of water used in aquaculture farms, poor nutritional status, and high stocking density. These parameters are also excessively influenced by interactions among the host, pathogens, and the kind of environment they are bred in = (Burge et al. 2014). A change in any one of these factors or climatic conditions can significantly affect the likelihood of an intense disease outbreak. (Burge et al. 2014). An acute concentration of contaminants and suspended particles can cause deformities and deaths in adult and seed fish (Kumari and Teacher 2020). Marine fish, particularly, are susceptible to various environmental challenges, such as toxins, and natural and biological intruders. These challenges are the primary risk factors for the prolonged suppression of immunity of marine aquatic species in the impacted marine environment which can lead to the occurrence of various bacterial infections (Olafsen 2001).

Numerous marine fish species worldwide, especially seabass (Vandeputte et al. 2019), suffer from tenacibaculosis, an ulcerative illness with significant mortality rates. *Tenacibaculum maritimum* is the primary causative agent of this disease. External clinical symptoms might include tail rot, superficial ulcerations, mouth erosion, and fin necrosis (Mabrok et al. 2023). Another one of the most common bacterial illnesses affecting various marine fish and shellfish is vibriosis. It can cause up to 50% of fish mortality and affects all stages of fish growth. The symptoms of this disease are seen as lethargic movement, skin ulcerations, fin rotting, loss of appetite, haemorrhage and congestion in liver, kidney, and spleen (Mohamad et al. 2019). Furthermore, overall systemic infections result in fish death (Deng et al. 2020). *Vibrio harveyi, Vibrio vulnificus, Vibrio parahaemolyticus, Vibrio alginolyticus*, and *Vibrio anguillarum* are a few of the *Vibrionaceae* species that are responsible for the disease. Freshwater and marine fish are susceptible to infection caused by an opportunistic bacteria *Mycobacterium marinum*. This infection results in morbidity and death in fish and necrotic granuloma resembling tuberculosis (TB). It is regarded as the most significant fish pathogen. It is linked to a variety of symptoms, including incoherent swimming, abdominal expansion, weight loss, skin ulcers, and the development of white nodules as granulomas in the liver, kidney, and spleen (Hashish et al. 2018). Among the highly recognized non-tubeculous mycobacterium (NTM) species linked to fish mycobacteriosis are *M. marinum*, *M. fortuitum*, and *M. chelonae*. About 200 species of marine and freshwater fish across an extensive range spanning from the subarctic zone to the tropical one are susceptible to the fatal piscine mycobacteriosis (Irshath et al. 2023).

The *Aeromonas* species are found to be quite prevalent among marine fish as seen in a study done by Yücel et al. 2010, where 97.3% of all their marine fish samples had been infected with *Aeromonas* species (Yücel and Balci 2010)*.* Among the species, *A. hydrophila* is a major cause of death in fish and shellfish (Aberoum and Jooyandeh 2010). Symptoms are seen as—haemorrhages and petechiae on internal and external organs, enlarged spleen, anorexia, and interstitial renal tissue necrosis (Menanteau-Ledouble et al. 2016). The Cytophaga-Flavobacterium (C-F) clusters cause the other less-known bacterial infections in marine fish. Fish that have been infected typically have acute necrotic lesions such as fin and tail rot, ulcerated skin, stomatitis, or jaw erosion and may even be septicaemic (Bernardet 1998). Further, *Aeromonas salmonicida*, known to affect salmonids in particular, causes lethal diseases, namely furunculosis which leads to severe septicemia, resulting in mortality especially in coldwater fishes. This disease is often marked by ulcerations in the dermal layer, subsequently leading to a septicemic condition along with haemorrhage (Cipriano and Bullock 2001).

Reared fish are subjected to high stress from intensive farming methods, which weaken their natural immune systems’ ability to fight off different bacterial and viral infections that can cause sickness. Regardless of whether a single species of fish is produced in dense populations or several, appropriate husbandry, and overall management, including biosecurity, nutrition genetics, system management, and water quality, are essential for their production.

## Current solutions

### Antibiotic administration

Antibiotic treatments have been widely used against bacterial illnesses in aquaculture for several years (Gram et al. 2001). According to Lulijwa et al (2020), around 73 per cent of the major aquaculture-producing nations use oxytetracycline, florfenicol, and sulphadiazine, while 55 per cent use erythromycin, amoxicillin, sulphadimethoxine, and enrofloxacin. They also noted that the use of antibiotics in Asian aquaculture output (constituting the largest producing nations), has increased by approximately 44.77 million tonnes between 2006 and 2016. Selective pressures from the usage of antibiotics fuel antimicrobial resistance (AMR). Over the past ten years, the availability of evidence around antimicrobial resistance has broadened (Buschmann et al. 2012; Schar et al. 2020; Thornber et al. 2022; Caputo et al. 2023).

A growing volume of research has connected AMR in marine fish with productivity loss and illnesses that are resistant to treatment, which has negative effects on fish and human health (Buschmann et al. 2012; Schar et al. 2021). The treatment options available in commercial rearing of marine fish may be reduced due to pathogen resistance, which could have an impact on food security and nutrition (Watts et al. 2017). The forthcoming sections of this review aim to elaborate on the developing resistance in pathogenic bacteria and high residue accumulation in the environment due to the incessant usage of antibiotics.

### Antibiotic resistance in pathogenic bacteria

Finfish aquaculture’s rapid expansion has led to several changes that are harmful to the environment and public health. The latter is demonstrated by the industry’s widespread and unrestricted use of prophylactic antibiotics, particularly in developing economies, to prevent bacterial illnesses brought on by unsanitary fish farming practises (Cabello 2006). The extensive use of antibiotics and the delayed progress in discovering and creating substitutes, such as vaccines and virulence inhibitors, are two of the fundamental issues with antibiotics (Griffin et al. 2020). More than 90 per cent of bacteria that developed in saltwater are multi-antibiotic resistant, and 20 per cent are at least resistant to a single antibiotic.

An examination of the presence of antibiotic resistance in native aquatic species is significant as it can reveal how human activity alters aquatic ecosystems (Baquero et al. 2008). Antimicrobial antibiotic accumulations in edible tissues can result in allergies and harmful effects, changes to the gut microbial fauna, and development of medication resistance (Ina-Salwany et al. 2019). *Aeromonas salmonicida* was the first fish pathogen to be identified as being resistant to sulphathiazol and tetracycline (Snieszko and Bullock 1957). Over time, many antibiotic resistance investigations have focused on *Vibrio* and *Aeromonas* because of their peculiar biofilm production and antibiotic resistance. Tetracycline, Streptomycin, and Kanamycin resistance genes were found in abundance in *Edwardsiella tarda*—a Japanese flounder (Yu et al. 2012). *Mycobacterium*, an important zoonotic fish pathogen that causes mycobacteriosis in both marine and freshwater fish, shows notable drug resistance (Guz et al. 2013). In a recent study, *Shigella spp.* isolated from saltwater fish were resistant to gentamicin, ciprofloxacin, and tetracycline 28 (17%) (Marijani 2022).

Studies have also demonstrated that human bacterial infections become resistant to antibiotics due to the exchange of antibiotic-resistance genes between bacteria in aquaculture and terrestrial environments. *Shewanella algae* and *Vibrio*, for instance, are aquatic bacteria that have been shown to cause antibiotic quinolone resistance in human Gram-negative pathogens (Ina-Salwany et al. 2019). In Japan and Chile, quinolone-resistance has been found in *V. parahaemolyticus*—an emerging human pathogen (Cabello 2006).

The World Health Organization (WHO) has declared that a number of antibiotics used in agriculture and aquaculture, including the antibiotic families tetracyclines, quinolones, and penicillin, are essential for human medicine (Done and Halden 2015). Many different bacteria, including those dangerous to humans, have been found to exhibit resistance to all antibiotic classes (Marijani 2022). Antibiotics in the aquatic environment may cause human pathogens that from the microbiota to develop resistance. The high level of contamination of seawater and freshwater with untreated sewage and agricultural and industrial wastewater containing normal intestinal flora and pathogens of animals and humans typically resistant to antibiotics in many aquaculture settings in developing countries has increased the possibilities of these exchanges. Large amounts of antibiotics entering and remaining in the environment of water and sediments in aquaculture have the potential to alter the presence of the typical flora and plankton in those niches, leading to changes in the diversity of the microbiota. This may have an impact on fish and human health by, for example, promoting algal blooms and anoxic environments (Cabello 2006). Table 1 is a collection of country-wise data on various diseases in marine fish, their causative microbes, and antibiotics used for their treatment. It depicts the extensive usage of prophylactic antibiotics across the globe, and highlights the reason for an upsurge in antimicrobial resistance.

**Table 1:** A summary of the country-wise division of various diseases in marine fish and their respective causative agents along with the antibiotics generally used for their treatment

Diseases	Fish Species Effected	Causative Microbes	Antibiotics used for treatment	Country	References
Vibriosis	Fin fish Salmon	*V. alginolyticus* *V. cholerae* *V. fluvialis* *V. hollisae* *V. anguillarum* *V. ordalii* *V. parahaemolyticus* *V. metschnikovii*	Ampicillin Carbenicillin Kanamycin Cefalothin	Italy Turkey Spain Algeria	Laganà et al. (2011); Ibrahim et al. (2020); Arab et al. (2020)
Enteric red mouth disease (ERM)	Salmonids	*Yersinia ruckeri*	Erythromycin Florfenicol Sulfonamid Trimetophrin	Turkey France	Onuk et al. (2019); Ibrahim et al. (2020)
Furunculosis	Salmonid Turbot	*Aeromonas salmonicida*	Sulfamerazine Nalidixic acid Oxytetracycline Ampicillin Amoxicillin, Ephalothin Erythromycin Streptomycin Sulfadiazine Trimethoprim Gentamicin Ofloxacin	Spain Turkey Italy Coratia	Ortega et al. (2006); Onuk et al. (2019); Ibrahim et al. (2020)
Hemorrhagic septicemia	Catfish	*Aeromonas veronii*	Sulfamerazine Nalidixic acid Oxytetracycline Ampicillin Amoxicillin, Ephalothin Erythromycin Streptomycin Sulfadiazine Trimethoprim Gentamicin Ofloxacin	Spain Turkey Italy Coratia	Ibrahim et al. (2020)
Rickettsial septicaemia	Salmon	*Piscirickettsia salmonis*	Tetracycline Trimethoprim Chloramphenicol Sulfamethizole	Canada	Rozas and Enríquez (2014); Shah et al. (2014); Saavedra et al. (2018)
Bacterial coldwater disease	Salmonids	*Flavobacterium* *psychrophilum*	Florfenicol oxytetracycline, chloramine T	Turkey Spain	Sekkin and Kum (2011); Saticioglu et al. (2018)
Bacterial kidney disease	Salmonids	*Renibacterium salmoninarum*	Erythromycin	United States of America Chile Iceland Japan	Sekkin and Kum (2011); Delghandi et al. (2020)

### Antibiotic residue accumulation

Antibiotic resistance has recently been identified as an emerging environmental issue (Griffin et al. 2019). Through excrement and leftover medicated feed, 75 per cent of the antibiotics used in pisciculture farms seep into the environment and build up as sediments. In a study by Lalumera et al (2004), priority chemicals for a monitoring programme in Italy examining potential environmental effects of the pisciculture industry included flumequine and oxytetracycline. Furthermore, Xiong et al (2015) detected concentrations of chlortetracycline, ciprofloxacin, doxycycline, enrofloxacin, norfloxacin, oxytetracycline, sulfametoxydiazine, sulfamethazine, and sulfamethoxazole in sediment and water samples at quantities of up to 446 μg kg^−1^ and 98.6 ng L^−1^, respectively. According to their investigation, the sediment and water samples contained a variety of taxa linked to opportunistic infections. Some of these genera, like Arcobacter and Treponema, are opportunistic human and animal diseases, whereas others, like *Clostridium* and *Acinetobacter*, are opportunistic human pathogens, causing diseases such as diarrhea and colitis. Since people who are frequently exposed to fish—such as consumers or those who work in the fish processing industry—are at high risk, the presence of pathogen-associated taxa in the fishponds could pose health risks.

Even subtherapeutic concentrations of antibiotics in aquatic environments have a key influence in the maintenance and enrichment of antibiotic resistance genes since most antibiotics were significantly accumulated in sediment samples of reservoirs in pisciculture environments (Cabello 2006). The use of antibiotics in aquaculture sectors is now subject to more stringent national laws and regulatory requirements because of the issue’s emergence as a public concern. Through a tripartite alliance between WHO, FAO, and The World Organisation for Animal Health (WOAH), formerly the Office International des Epizooties (OIE), the WHO adopted the "One Health Strategy" to address the ABR problem worldwide. In collaboration with its alliances, the WHO released the "Global Action Plan (GAP) on AMR" in 2015 (*WHO Library Cataloguing-in-Publication Data Global Action Plan on Antimicrobial Resistance*, 2015). Many nations, including Japan, the United States, and Colombia, as well as the EU’s Denmark, the Netherlands, and Sweden, have put in place national objectives to minimize antibiotic use, as well as benchmarks for antibiotic use at the farm level and antibiotic stewardship (Walia et al. 2019). Furthermore, The Progressive Management Pathway for Improving Aquaculture Biosecurity (PMP/AB) also focusses on enhancing disease prevention at the farm level by ethical fish farming (including lowering antimicrobial resistance in aquaculture and applying appropriate antimicrobial alternatives) and other science and technology-based strategies (“The State of World Fisheries and Aquaculture 2020,” 2020).

If the usage of antibiotics is inevitable due to rapidly spreading critical illness, it should have effective control and monitoring protocols to minimize the concerns outlined above. Alternative and more sustainable ways can be applied through various farming practices, such as optimizing water quality (*RAS Roundtable: Feeding Strategies to Maintain Optimal Water Quality and Fish Performance*) and reducing stocking densities to reduce stress and disease risk (Mohanty et al. 2018). The sustainable alternatives further increase customer knowledge and demand for ecologically and responsibly farmed seafood, which can affect marine fish rearing and disease management practices. Consumer demand for antibiotic-free fish, as well as certification of sustainable farming practices, can encourage farmers to use more ecologically friendly and antibiotic-free methods. The notion of a large marine ecosystem seeks to manage oceans sustainably. Apropos this, detection and treatment of emerging diseases becomes imperative, with focus on sustainable disease management techniques.

### Phage treatment

A bacteriophage is a virus that grows and divides inside a bacterium, destroying it.. It consists of proteins that encase a DNA or RNA genome and replicate inside the bacterium after the genome is injected into the cytoplasm (McGrath et al. 2004). Seawater is one of the most abundant natural sources of bacteriophages and other viruses (Keen 2012). Unlike terrestrial animals, pisciculture species and their surrounding environment can be subjected to bacteriophages to simultaneously remove diseases in the organism and its immediate habitat. Therapeutic results can be influenced by treatment plans, which include the quantity and frequency of bacteriophages used as and their method of administration (Richards 2014). Many experimental studies of experimental in vivo phage treatments focusing on *Vibrio, Aeromonas, Pseudomonas, Acetobacter, and Flavobacterium* have previously demonstrated the efficacy of bacteriophage therapy in marine fish (Park et al. 2000; Laanto et al. 2015; Silva et al. 2016; Assefa and Abunna 2018).

Studies on fish immunity, however, have only infrequently documented the specific interactions between members of the genera *Vibrio* and *Edwardsiella* and bacteriophages (Ramos-Vivas et al. 2021). By minimising the loss of phage activity, phage-loaded edible whey protein isolate coatings improve fish therapy. According to the results of a simulated testing for gastric-intestinal digestion, this technique increases phage stability while lowering bacterial levels. Additionally, it enables the regulation of phage release in seawater and safeguards them until they get to their intended target (Huang and Nitin 2019). The solution to a positive outcome is believed to be early therapy (Ramos-Vivas et al. 2021).

Phage therapy may be a good substitute for antibiotics in the treatment of fish pathogenic bacteria, but it must be used with knowledge of kinetics phenomena. Silva et al (2014) conducted a study to determine the impact of the physical and chemical characteristics of aquaculture waters—pH, temperature, salinity, and organic matter content—on the effectiveness of phage therapy in carefully controlled experimental settings. The observed that the fluctuation of salinity and organic matter concentration had the greatest influence on the effectiveness of phage therapy. Phage therapy appeared as a good option for marine aquaculture systems because its efficacy rises with the salt concentration of the water. When salt addition is a feasible choice and does not negatively impact the survival of aquatic organisms being grown, phage therapy may potentially be more effective in non-marine environments. Furthermore, they also noticed that the native bacterial populations in aquaculture waters should not be significantly affected by the bacteriophages’ ability to inactivate harmful bacteria.

### Vaccine administration

In the Norwegian fish farming business, for example, antibiotic use has reduced by more than 90% since the 1980s. Grave et al. (1996) described how bacterial illnesses such as vibriosis and cold water vibriosis generated a significant increase in the usage of antibacterial agents in Norwegian aquaculture during the growth of industrialized salmon farming in the 1980s. The quick drop in antibiotic consumption was due to the cooperative efforts of the Norwegian fish farming sector and the Norwegian government in researching and promoting fish vaccine immunization procedures (Grave et al. 1990). This demonstrates that large-scale production of farmed fish is viable without the use of antibacterial medications on a regular basis, which is a crucial factor for the sustainability of any industrialised fish production system. However, fish vaccination also has its drawbacks. According to a study by Ma et al (2019), vaccinations include or create a material called an antigen that causes an aquatic creature to mount an innate or adaptive immune response against a specific pathogenic organism. The immune reaction defends against illness and fends off upcoming illnesses. Only in the mid-1970s was there more interest toward using vaccines in fish farming to boost the hosts’ ability to fight illness. Since then, there have been myriad studies over the course of 1970 to 1990 and up wards on different species of fish (Amend et al. 1983; Braaten and Hodgins 2011; Cipriano 2011; Cipriano and Starliper 1982; Michel 1979; Paterson and Fryer 2011; Thune and Plumb 1982). There are several different vaccinations that have previously been created, including bacterin, toxoid, DNA, subunit, live attenuated, whole-cell, and anti -idiotypic vaccines. Currently, killed whole-cell vaccines are the most widely utilized commercially available and approved vaccinations in the aquaculture sector. Other vaccinations are being created; however, they are either still in the experimental or clinical trial stages (Mohd-Aris et al. 2019).

During the past twenty years, several nucleic acid vaccines have been created for use in aquaculture. These vaccines have been said to combine the beneficial qualities of both live attenuated and subunit vaccinations (Ulmer and Geall 2016). Subunit vaccines can tailor immune responses against certain microbial determinants and allow the insertion of unnatural components. Since subunit vaccines cannot reproduce in the host, there is no possibility of pathogenicity to the host or non-target species. Subunit vaccines benefit from employing just antigenic components for immunisation (Hansson et al. 2000; Holten-Andersen et al. 2004). Reviews of the effects and applications of DNA vaccines have been published several times (Kurath 2008; Hølvold et al. 2014; Dalmo 2018). In most fish species, including those of marine origin, these vaccines are administered via intramuscular (IM) injection since the genetic material must be reasonably protected to enter host cells (Heppell et al. 1998).

Finding the ideal bacterial strain is essential to produce a vaccine, but there are many challenges to overcome because of different serotypes which has slowed the development of a vaccines against bacterial infections (Ina-Salwany et al. 2019). Standardized in vivo disease challenge models that closely resemble the pathogen’s natural exposure pathway are necessary to test the effectiveness of vaccinations. Furthermore, the variety of fish species themselves is a hurdle for vaccine development since each fish species needs reagents or primers to help explain host–pathogen interactions. Additionally, although injection is frequently used for marine fish such as the Atlantic salmon, it might not be practical for certain species, such as pangasius. There is also a dearth in efficient commercial immersion adjuvants for fish (Adams 2019).

Problems that impede the creation of multivalent, affordable vaccination programmes have not yet been overcome (Mondal and Thomas 2022). Adverse side effects following vaccination are another major constraint in fish vaccines regime (Ina-Salwany et al. 2019). Additionally, while there are various modes of vaccine delivery, one of which includes utilizing modified live vaccines via an active, viable pathogen, the method has significant safety drawbacks due to the possibility of insufficient vaccine death (Shoemaker et al. 2009). Another issue commonly faced is the challenges with respect to the usage of unlicensed vaccines and their abundance in the market. Furthermore, a systematic evaluation of their effectiveness against local strains is understudied (Sommerset et al. 2005).

### Biosurfactants

Microorganisms naturally create surface-active chemicals known as microbial surfactants or biosurfactants (BS). They include both hydrophilic and hydrophobic components, such as acids, peptides, mono-, di-, and polysaccharides, and saturated and unsaturated hydrocarbon chains and fatty acids (Rodríguez-López et al. 2019). The amphipathic character of BS causes them to cluster at surfaces and lower interfacial tension, enhancing the solubility of hydrophobic chemicals in the water.

Currently, BS are an essential component in various industrial applications (Giri et al. 2020). BS have been investigated to strengthen marine fish defence systems against different infections and have been employed as immunostimulants in marine fish production (Giri et al. 2020).

In a study by Hamza et al (2017)*, Vibrio harveyi* and *Pseudomonas aeruginosa* biofilms were inhibited by the biosurfactant (at a concentration of 20 µg) by 80.33 ± 2.16 and 82 ± 2.03%, respectively. At this concentration, it was also effective at dislodging mature biofilms of *P. aeruginosa* (81.7 ± 0.59%) and *V. harveyi* (78.7 ± 1.93%). Ibacache-Quiroga et al (2013) focussed on a study where the fish pathogen *Cobetia sp.* strain MM1IDA2H-1 created a biosurfactant that disrupted *Aeromonas salmonicida’s* ability to sense quorums through signal hijacking Giri et al (2017) noted that *Bacillus licheniformis* VS16-derived biosurfactant proved successful in preventing the growth of biofilms in *Aeromonas hydrophila* up to 54.71 ± 1.27%. In a study by Rajeswari et al. (2016), *O. Mossambicus* was intraperitoneally injected with different amounts of a water-soluble secondary metabolite (biosurfactant) of *Staphylococcus hominis*, and the results showed a boost in immunity along with facilitating a potential solution toward the pisciculture industry’s development. The impact of feeding poly-β-hydroxybutyrate (PHB)-enriched *Artemia nauplii* on the immunological responses and survival of post-larvae of the European sea bass (*Dicentrarchus labrax*) was examined by Franke et al (2017). The expression of the relative growth indicator insulin-like growth factor 1 (Igf1) was elevated, despite the survival of the larvae being unchanged. Large yellow croakers fared better when PHB was used as a feed supplement, according to Wang et al (2019).

The market for biosurfactants is underdeveloped because of the excessive cost of substrates, downstream processing, and biosurfactant yield. Purifying products with the same polarity against organic solvents is difficult and expensive, which is the primary limitation to the product recovery process. Additionally, biosurfactant screening procedures are time-consuming and labour-intensive (Gaur et al. 2022).

## Current antimicrobials and other alternative solutions

With the issues associated with antibiotics as the existing prophylactic or therapeutic agents and vaccines, being available only for a limited number of species of fish, there is an increasing shift towards the use of antimicrobial peptides (AMPs) due to their antimicrobial and immunomodulatory properties (Colwell and Grimes 1984; Haney and Hancock 2013).

The mechanism of these antimicrobial peptides depends on several things, including but not limited to the sequence of the amino acids, their charge, amphipathic property, the secondary structure (Kumar et al. 2018). The mechanism of AMPs from various sources has been extensively studied. There appear to be two major mechanisms causing the antimicrobial activity—direct killing and immune modulation. The direct killing mechanism of action can be divided further into membrane targeting and non-membrane targeting. The membrane-targeted approach could either be receptor-mediated or non-receptor mediated (Kumar et al. 2018).

Most AMPs derived from vertebrates and invertebrates have non-receptor-mediated mechanisms of action. For the cationic ones, the difference in their charge and that of the negatively charged membrane of Gram-negative bacteria helps initiate the interaction. The mammalian cells do not contain anionic molecules oriented pointing outwards of the cell, making the peptides selective (Epand and Vogel 1999). However, it has been observed in some cases that there may not necessarily be a relation between membrane perturbation and the antimicrobial activity of the peptide and that it may just be an enabler for the peptide to reach its actual target inside the cell (Wu et al. 1999).

Other mechanisms that may be involved in inducing antimicrobial activity interfere with metabolic processes—inhibition of cell walls, and proteins, nucleic acid, or enzyme synthesis, consequently making it difficult for the bacteria to develop resistance against them (Pletzer and Hancock 2016).

### Antimicobial peptides

**Fig. 2:**
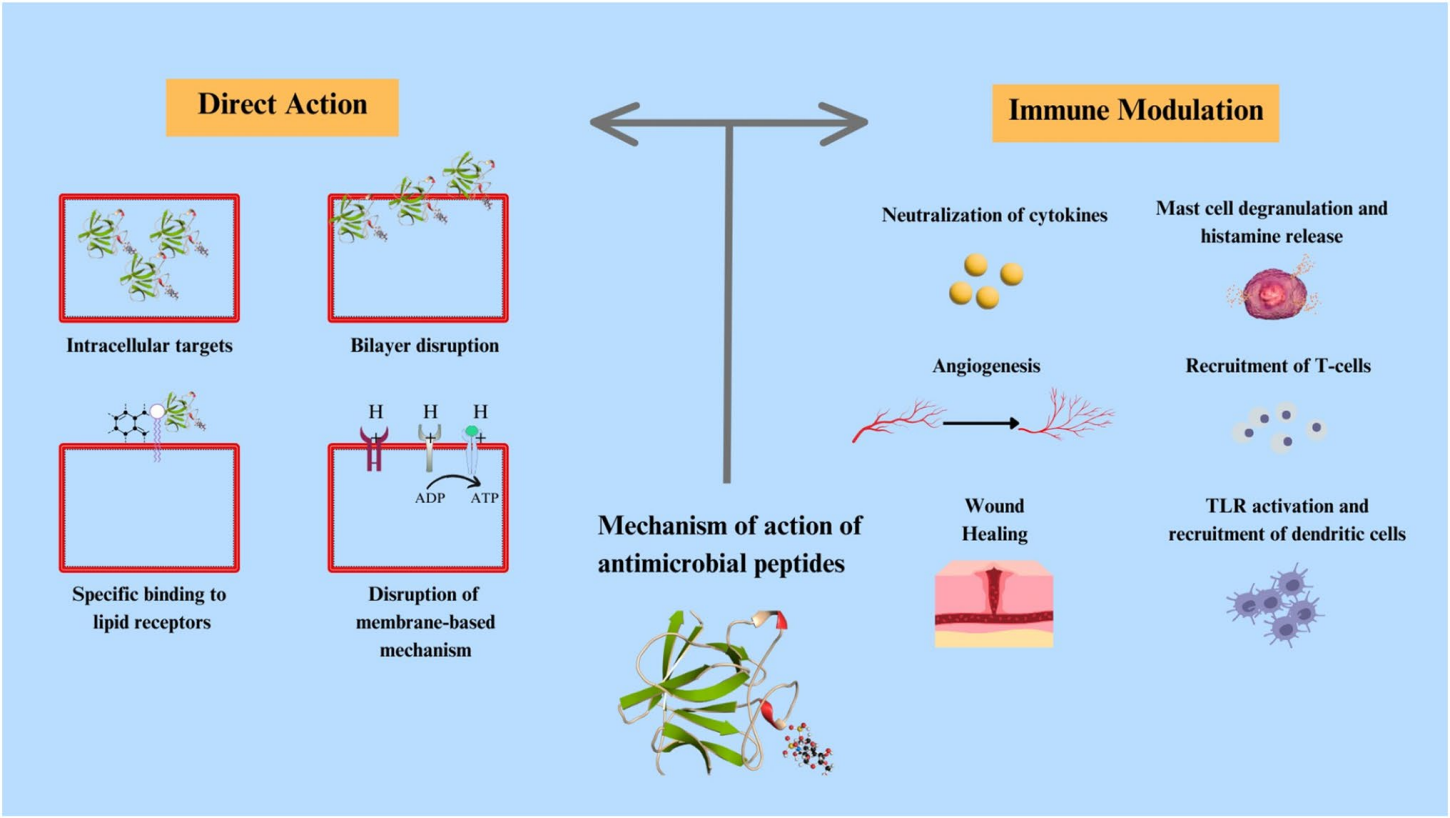
A schematic representation of the mechanism of action of antimicrobial peptides highlighting their direction action and methods of immune modulation. (Kumar et al. 2018; Singh et al. 2022)

Antimicrobial peptides form a part of the innate host defence system in various organisms, including fish. Figure 2 depicts their mechanism of action—direct and immune modulation strategies are employed by these peptides. As discussed above, they have a broad-spectrum activity against variety of pathogens, remain potent under various conditions, including extremities of temperature, saliva, marine environments, and possess a low capacity to develop resistance against bacteria (Cole et al. 1997a). This creates a plethora of opportunities to develop naturally produced peptides into therapeutic agents. Thus, a detailed analysis of the structure, function, and activity of various kinds of pathogens is necessary.

There are five major known classes of peptides—Piscidins, Defensins, Hepcidines, Cathelicidins, and Histone-derived peptides. Table 2 is a detailed representation of them alongside their corresponding antibacterial properties.

**Table 2:** A compilation of the peptides alongside their corresponding antibacterial properties with focus on marine habitats (Masso-Silva and Diamond 2014)

Organism	ꞵ-Defensins	Piscidin	Hepcidin	Cathelicidin	Histone-derived	Habitat
Bacteria- gram negative						
*Aeromonas salmonicida*	> 50 μM (MIC) Ruangsri et al. (2013)	17.7–35 μM (MIC) Cole et al. (1997b)	> 44 μM (MIC) Lauth et al. (2005)	9.38 μg/mL (MIC) Li et al. (2013)	20 μg/mL (MIC) Birkemo et al. (2003)	Freshwater and marine
*Vibrio anguillarum*	> 50 μM (MIC) Ruangsri et al. (2013)	(Strain- E-3–11) 20–40 μM (MIC), (strain- SGL8542) 40–80 μM (MIC) Sun et al. (2007)	2.92 μM (MIC) Wang et al. (2012)	10–20 μg/mL (MIC) Bridle et al. (2011)		Marine
*Vibrio parahaemolyticus*			> 60 μM (MIC) Yang et al. (2011)	25μg/mL (MIC) Lu et al. (2011)		Marine
*Vibrio fluvialis*	43.0 ± 10µg/ml (vLD90) Jin et al. (2010)		> 96 μM (MIC) Qu et al. (2013)	3.125μg/mL (MIC) Lu et al. (2011)		Marine
*Vibrio harveyi*		12.5μg/mL (MIC) Pan et al. 2007)	20–40μg/mL (MIC) Yang et al. (2011)	6.25μg/mL (MIC) Lu et al. (2011)		Marine
*Vibrio alginolyticus*		> 23.57μg/mL (MIC), > 19.52μg/mL (MIC), 2.44μg/mL (MIC), 0.03μg/mL (MIC), > 17.64μg/mL (MIC), 1.24μg/mL (MIC), 2.68μg/mL (MIC) Peng et al. (2012)	> 60 μM (MIC) Yang et al. (2011)			Marine
*Vibrio vulnificus*		> 23.57μg/mL (MIC), > 19.52μg/mL (MIC), 2.44μg/mL (MIC), 0.03μg/mL (MIC), > 17.64μg/mL (MIC), 0.62μg/mL (MIC), 0.67μg/mL (MIC) Lauth et al. (2002)	50μg/mL and 400μg/mL (MIC) Zhou et al. (2011)			Marine
*Vibrio cholera*		2.5–5 μM (MIC) Lauth et al. (2002)				Freshwater, marine and others
*Pseudomonas aeruginosa*	44.5 ± 11.8µg/ml (vLD90) Jin et al. (2010)	> 23.57μg/mL (MIC), > 19.52μg/mL (MIC), > 19.55μg/mL (MIC), 0.52μg/mL (MIC), > 17.64μg/mL (MIC), > 19.78μg/mL (MIC), 10.70μg/mL (MIC) Peng et al. (2012)	> 44μg/mL (MIC) Lauth et al. (2005)	12.5μg/mL (MIC) Lu et al. (2011)		Freshwater, marine and others
*Edwardsiella tarda*			2.92μM (MIC) Wang et al. 2012)			Marine and freshwater
*Edwardsiella ictaluri*				25μM (MIC) Lu et al. (2011)		Freshwater and marine
*Edwardsiella piscicida*		73.8 μM (MIC) Simora et al. (2021)				Freshwater and marine
*Flavobacterium coulumnare*		> *200* µg/ml (MIC) Simora et al. (2021)				Freshwater and marine
Bacteria- gram positive						
*Streptococcus agalactiae*		> 23.57μg/mL (MIC), > 19.52μg/mL (MIC), 9.78μg/mL (MIC), 2.10μg/mL (MIC), > 17.64μg/mL (MIC), 9.89μg/mL (MIC), > 21.41μg/mL (MIC) Peng et al. (2012)				Freshwater, marine and others
*Streptococcus iniae*		3.1μM (MIC), 25.0μM (MIC), 12.5μM (MIC) Noga et al. (2009)		> 100μM (MIC) Lu et al. (2011)		Freshwater, marine and others
*Mycobacterium smegmatis*		75μg/mL (MIC) Cole et al. (2000)				Freshwater and marine

### Synthetic peptides

Albeit several studies have been performed using natural AMPs using their antibacterial, antifungal, and antiparasitic properties (Jia et al. 2000; Chettri et al. 2017), there continue to exist inherent disadvantages associated with natural AMPs such as instability due to presence of proteolytic sites and high manufacturing cost (Hancock and Scott 2000).

Thus, there has been a shift towards designing and manufacturing more stable, shorter peptides with higher efficiency. RY12WY is a novel peptide designed based on a knowledge-based approach and considering the various physiochemical properties. Unlike natural amino acids, it is shorter and contains only hydrophobic, positively charged amino acids. It has shown antimicrobial activity against *S. aureus, A. hydrophila,* and *A. salmonicida*, known antibiotic-resistant fish pathogens. It also showed activity against *E.coli and S.parasitica* (Hussain Bhat et al. 2020).

Recently, the 2022 iGEM Team of MIT_MAHE (AMPIFIN | MIT_MAHE-IGEM 2022) designed an antimicrobial peptide—AMPifin—against *V. parahaemolyticus.* The peptide was designed based on the interaction between the membrane protein Multivalent Adhesion Molecule 7 (MAM7)—present on the surface of the bacteria and the host cell ligands. Despite the several advantages of antimicrobial peptides as therapeutics, some species of bacteria have developed resistance against them. The bacteria employ any of the various mechanisms—bacterial cell envelope modification, bacterial proteins degrading or sequestering the peptides, and expelling of the AMPs (Abdi et al. 2019). They may also require additional delivery mechanisms to account for their instability, easy degradation by proteases, and to work effectively (Martin-Serrano et al. 2019). These reasons make the application of antimicrobial peptides as therapeutic agents difficult.

### Probiotics

The idea of using probiotics or other beneficial microorganisms as a disease bio-control strategy in aquaculture is based on their advantageous roles in improving water quality, regulating fish health, altering the microbial community in the aquatic environment and within the GI tract, and promoting non-specific immune response and resistance against pathogens (Li et al. 2019), which have positive effects on growth performance and nutrient utilisation (Martínez Cruz et al. 2012).

**Fig. 3:**
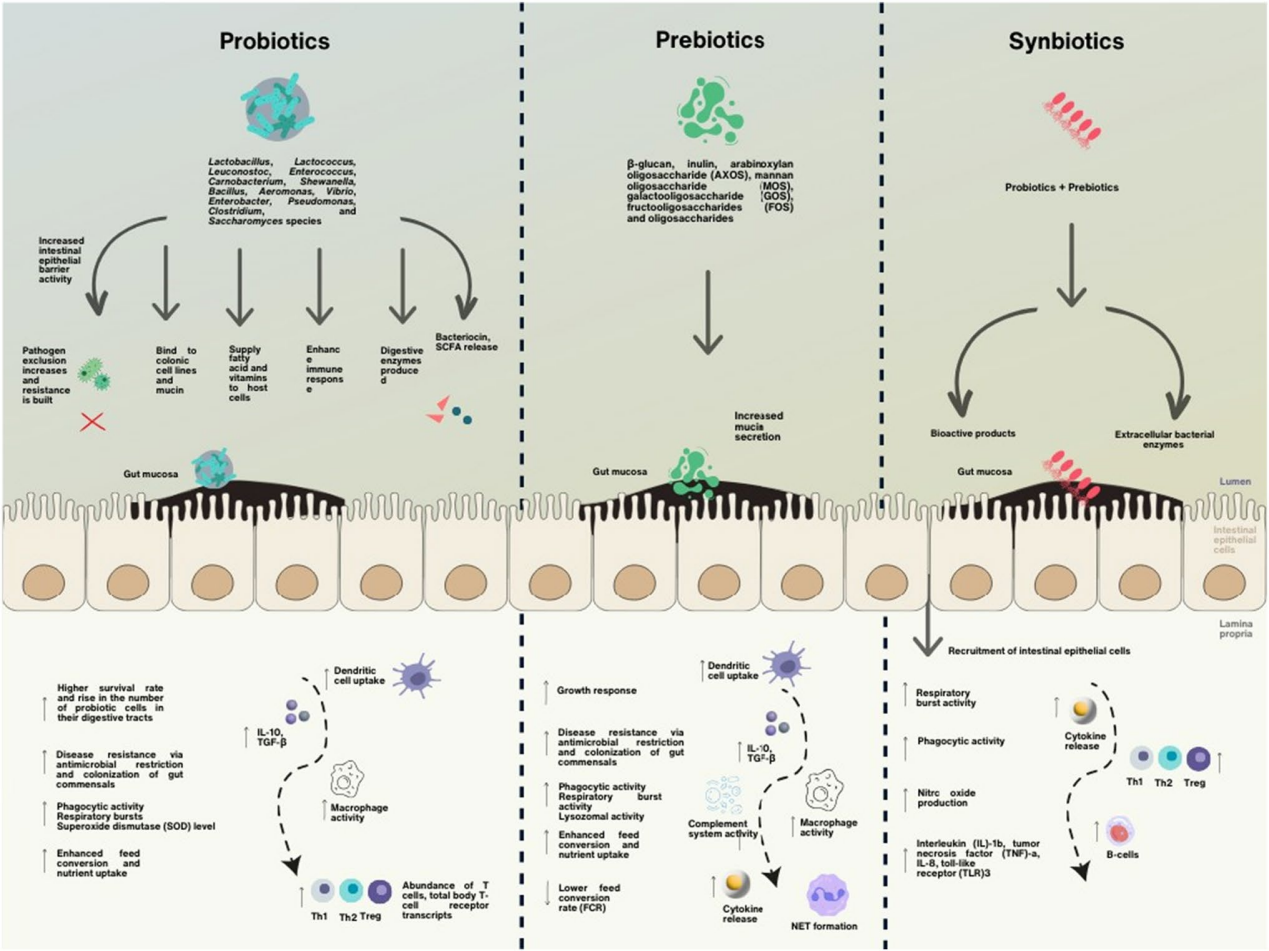
Probiotic, prebiotic, and synbiotic therapeutics’ mechanisms for conferring pathogen resistance and enhanced immunity in fish. (Talukder Shefat 2018; Wuertz et al. 2021; Dawood and Koshio 2016; Huynh et al. 2017; Nayak 2010; Wee et al. 2022)

These advantageous bacteria can colonise and grow in the gut of the host following injection and carry out a variety of positive effects by modifying the host’s biological mechanisms (Skjermo and Vadstein 1999; Nayak 2010; Akhter et al. 2015). Probiotics’ general mode of action involves a probiotic organism colonising the gut and preventing harmful bacteria from doing the same. Pathogenic organisms are hampered by specific inhibitory compounds produced by probiotic organisms. Then, probiotic organisms use the resources, making them unavailable to pathogens. Probiotics create substances that work against the quorum sensing system, and increased macrophage activity and antibody levels improve immunity. Probiotics can increase the digestibility of feed, the net availability of critical nutrients, and the host animals’ immunity and gastrointestinal health (Rohani et al. 2022). Figure 3 is a schematic representation of the mechanism of action with respect to the usage of probiotics in fish.

In trials using probiotics as dietary supplements, many metrics, including weight gain, specific growth rate, feed conversion ratio, and protein efficiency ratio, are routinely evaluated to examine changes in growth and feed efficiency (El-Dakar et al. 2007; Jahan et al. 2021; Putra et al. 2017; Rohani et al. 2022). In addition to enhancing feed digestion, probiotics also help larvae absorb and use nutrients from their yolks before their first meal, which is one way they help probiotics increase feed digestion (Irianto and Austin 2002a). Probiotics are known to improve epithelial barrier function through their interaction with toll-like receptors (TLRs), which stimulates cytokine production and starts innate and adaptive immune responses in the host body, even though the immunomodulatory effects of probiotics are not fully understood (Pillinger et al. 2022). To augment innate immune responses, probiotics interact with immune cells such as mononuclear phagocytic cells (monocytes, macrophages), polymorphonuclear leucocytes (neutrophils), and NK cells. Some probiotics can increase the number of erythrocytes, granulocytes, macrophages, and lymphocytes in certain fish, similar to higher vertebrates (Irianto and Austin 2002b; Kumar et al. 2008; Kuebutornye et al. 2019; Kong et al. 2020; Rohani et al. 2022; Zhu et al. 2023). Table 3 is a summary of recent studies in marine fish with varied experimental designs to test antioxidant status, immunity, and disease resistance post probiotic administration. Their activity may be explained by the presence of a special probiotic component, such as β-glucan in yeast cell walls. Probiotics also contain specific phagocytic cells receptors that help to bind receptors molecules on the cell surface of the phagocyte, which will help to release signal molecules and ultimately stimulate the production of new WBCs.

**Table 3:** A summary of marine fish—antioxidant status, immunity, and disease resistance following administration of several probiotic species and strains

Probiotic species	Fish species	Experimental design	Period	Challenge	Effects compared to control	References
*Shewanella putrefaciens* (Pdp11)	Gilthead Seabream	20 kg/m^3^ of high stocking density feed with 1011 CFU/kg	28 days	N/A	Low phagocytic capability; high head renal leucocyte and cellular peroxidase activity; IL-6, TGF and IgM levels did not alter	Cordero et al. (2016)
*Bacillus velezensis*	European Sea Bass	10^6^ CFU g^−1^ of feed	30 days	*Vibrio anguillarum*	Boosted the innate humoral activities without causing any discernible negative physiological changes	Monzón-Atienza et al. (2022)
*B. thuringiensis* QQ1 *B. cereus* QQ2	Asian Seabass	10^12^ CFU/kg diet	35 days	*Vibrio harveyi*	High cumulative survival after a challenge and high RBA	Ghanei-Motlagh et al. (2021a, b)
*B. subtilis E20*	Grouper (*Epinephelus coioides*)	10^7^, 10^9^ and 10^11^/kg	28 days	*Streptococcus sp.*	High phagocytic activity, respiratory bursts, and SOD level of head kidney leucocytes as well as serum lysozyme activity	Liu et al. (2012)
*L. delbrueckii sp.* (AS13B)	European seabass (*Dicentrarchus labrax*)	10^5^ bacteria/cm^3^	75 days	N/A	Intestinal mucosa had an abundance of T cells, total body TcR- transcripts, and acidophilic granulocytes	Picchietti et al. (2009)
*Lactococcus lactis BFE920*	Olive flounder	1 × 10^9^, 5 × 10^9^, 2.5 × 10^10^ and 1.25 × 10^11^ CFU/kg feed	14 days	*Streptococcus iniae*	High Lysozyme, IL-12, IFN- γ, and MP activity, as well as a high SR% in challenged fish	Kim et al. (2013)

*IFN-γ* Interferon gamma, *Igs* Immunoglobulins, *IL-6* Interleukin 6, *IL-12* Interleukin 12, *MP* Macrophage, *RBA* Respiratory bust activity, *SOD* Superoxide dismutase, *SR* Survival rate, *TcR* T-cell receptor, *TGF-β* Transforming growth factor beta

It has been reported that multi-strain probiotics are more effective than single-strain probiotics at preventing illness (Vazirzadeh et al. 2020), and they have been suggested for use in aquaculture (Melo‐Bolívar et al. 2021). Such advice is justified by the idea that combining different microbes will synergistically affect the host’s health. However, only a few studies have compared the efficacy of multi-strain probiotics with that of each individual strain in their composition, and even fewer studies have investigated their impact on fish immune, particularly when confronted with pathogens. The technique of introducing these diverse feed additives is not easy or simple. In addition to adding to costs, new feed additives also require attention to ensure that novel microbial strains are applied safely and as effectively as possible. To prevent potential injury or unfavourable side effects, strict restrictions should be put in place to create suitable procedures of manufacture and application of these chemicals (Ayisi et al. 2017). Probiotics are also known to play a major contributing factor in the decomposition of organic matter, reduction of nitrogen and phosphorus levels as well as control of ammonia, nitrite, and hydrogen sulfide (Kim et al. 2012).

In a recent study, the development, survival, and innate and adaptive immune systems of cobia fish (*Rachycentron canadum*) were all greatly improved by the combination of autochthonous strains, *Bacillus sp.* RCS1, *Pantoea agglomerans* RCS2, and *Bacillus cereus* RCS3 (Amenyogbe et al. 2022). In recent work, Paul et al. (2022) focussed on uncovering native autochthonous bacteria from the catfish gut and assessing their effects on *Aeromonas veronii* infection in *Clarias batrachus*, a freshwater fish and *Heteropneustes fossilis*, a marine fish in a lab setting. All fish treated with probiotics had faster growth rates than controls. Both *Lactobacillus sp.* and *Bacillus sp.* considerably (p < 0.05) increased growth and survival in catfish, with *Lactobacillus sp.* Having the strongest benefits in *H. fossilis* and *C. batrachus*, respectively. Fish treated with probiotics showed higher survival rates after intramuscular (IM) injection with *A. veronii,* according to the study. Additionally, recent studies discovered that “autochthonous” or host-derived probiotics may also be employed as quorum quenching probiotics, conferring health advantages, enhancing defensive systems, and safeguarding fish against vibriosis (Ghanei-Motlagh et al. 2021b). Autochthonous probiotics, or species-specific strains that are suited to the intestinal microhabitat of the cultivated target species, must be isolated and developed. Because it may enhance the growth efficiency, immune system, and defense against illnesses in situ, the isolation and selection of native strains drives the development of the production of native species. There seems to be a broad opinion that lactic acid bacteria strains (LABs) are more likely to have the qualities and features required to colonize the gut and promote host health among the autochthonous probiotics (Yamashita et al. 2020).

In addition to probiotics, there is also ongoing research on prebiotics, which are usually expected to improve the number of beneficial gut microbiota in the form of indigestible fibres. This improves innate immunity by increasing the phagocytic cell activation, augmenting lysozyme activity and activating the alternative complement system in fishes (Yilmaz et al. 2022). They inhibit the adhesion of organisms to the organism by competing for the same glyco-conjugates, increasing mucus production, inducing cytokine release and producing short chain fatty acids (Cavalcante et al. 2020).

Furthermore, a synergistic amalgam of prebiotics and probiotics that are usually in the form of live cells of beneficial microbes and a selective substrate (Rohani et al. 2022). They positively effect the health and welfare by implanting live microbial supplements in the host’s gastro-intestinal tract and thus increasing survival rates (Yilmaz et al. 2022). They also sometimes improve the feed digestibility and total intestinal enzyme activity which enhances the growth performance of the host (Rohani et al. 2022).

### Medicinal plants

Medicinal plants were introduced as a viable and alternative strategy for treating fish sickness due to the negative effects of veterinary pharmaceuticals used in aquaculture, either on fish or the environment and human health. Indeed, due to their abundance of minerals and chemical components, medicinal plants are utilised in aquaculture not only as chemotherapeutics but also as feed additives (Chang 2000; Wang et al. 2015). Marine fish growth enhancement, hunger stimulation, immunological stimulation, antibacterial properties, and stress reduction have all been linked to medicinal herbs (Chitmanat et al. 2005; Citarasu 2010; Chakraborty and Hancz 2011). Medicinal plants can modulate the innate immune system by enhancing the protease inhibitors and lytic enzymes of immune cells and molecules to react against the invading pathogen (Sakai 1999; Van Hai 2015).

Several studies have reported the enhancement in immunological parameters in many species after administration of medicinal plants or extracts including phagocytic activity, respiratory burst activity, nitrogen oxide, myeloperoxidase content, complement activity, lysozyme activity, total protein (globulin and albumin) and antiprotease activity (Dügenci et al. 2003; Wu et al. 2010; Talpur and Ikhwanuddin 2012; Talpur 2014). Traditional medicinal plant items also provide immunomodulation, defence against bacterial infections, and suppression of infections. With the use of *Solanum nigrum*, it was discovered that spotted snakeheads’ resistance to *Aeromonas hydrophila* infections was boosted, and their death rate was decreased (Rajendiran et al. 2008). According to Harikrishnan et al (2012), *Epinephelus bruneus* had improved defences against *Vibrio harveyi* when fed kudzu vine Moreover, extracts of mango, peppermint, turmeric, jasmine, neem, and other plants are among the other effective treatments for bacterial infections in aquatic species brought on by *Aeromonads* and *Vibrios* (Newaj-Fyzul and Austin 2015). Wang et al (2011) investigated the effects of adding polysaccharides from *Angelica sinensis* (0.5 and 3 g kg^−1^) to the *Epinephelus malabaricus* diet. Both immunological parameters and disease resistance were assessed at the conclusion of the feeding trial, and the results showed that cellular immunity had been stimulated and there was greater protection against *Edwardsiella tarda* (Wang et al. 2011). Pan et al. (2013) examined the effects of 20 g/kg of the medicinal plant *Astragalus membranaceus* on red drum (*Sciaenops ocellatus*) and found that it increased immunological parameter activation and resistance to *Vibrio splendidus.*Asian sea bass (*Lates calcarifer*) fingerlings were used in a study by Talpur and Ikhwanuddin (2012) to examine the effects of different amounts of garlic supplement (5, 10, and 20 g kg^−1^) on immunological parameters and resistance to *V. harveyi*. Results showed that Asian sea bass given garlic had significantly higher levels of immunological parameters and a higher survival rate after being challenged with *V. harveyi* (Talpur and Ikhwanuddin 2012). Table 4 is a summary of recent studies in marine fish with varied experimental designs to test antioxidant status, immunity, and disease resistance post medicinal plant extract administration.

**Table 4:** The table is a compilation of recent studies where medicinal plants have been administered to study their immunomodulatory and antibacterial effect on fish

Medicinal plant species	Experimental bacterial species (challenge)	Experimental fish species	Parameters	Method of administration	Effects on fish	References
*Allium sativum* (garlic)	*A. hydrophila*	*Oncorhyncus mykiss*	0.5 and 1.0 mg/g feed; 14 days	Oral—through feed	Reduction in mortality by 4%; enhancement of bactericidal activities	Vaseeharan and Thaya (2014)
*Astragalus membranaceus r*oot + *Angelica sinensis* root	*Vibrio alginolyticus*	*Pseudosciaena crocea*	0.5%, 1.0% and 1.5% (w/w) mixture of the roots; 20, 25, and 30 days	Medicated diet via feed	Enhanced lysozyme and complement activities; NBT-positive cells as well as survival rate (93.3%)	Maqsood et al. (2011)
*Terminalia chebula, Polyalthia longifolia, Terminalia bellerica*, and *Phyllanthus emblica*	N/A	*Aeromonas hydrophila (MTCC 646)* and *Pseudomonas fluorescens (MTCC 103)*	Concentration: 2,000 µg/ml of extract	Antibacterial activities were evaluated by measuring the inhibition zone diameters	Minimised the presence of the pathogen	Ghosh et al. (2011)
*Nigella sativa* (black cumin seed)	N/A	*Oncorhyncus mykiss*	Lyophilized extracts of 0.1 and 1% was used at a rate of 2% of body weight; 3 weeks	Oral—through feed	Increase in intracellular activity	Dügenci et al. (2003)
Fresh leaves of *Azadirachta indica Juss* (Meliaceae)	*Pseudomonas aeruginosa, Streptococcus* sp	*Amphiprion sebae*,* A. ocellaris*	Concentration: 500 µg of crude extract; 18 h	Antibacterial activities were evaluated by measuring the inhibition zone diameter	Significantly reduced the bacterial population in fish	Dhayanithi et al. (2010)
*Zingiber officinale*	*Vibrio harveyi*	*Lates calcarifer*	Dosage: 0.55 and 10 g/kg feed; 15 days	Oral—through feed	RBC (erythrocyte), neutrophil, and WBC count increased	Talpur et al. (2013)
*Mentha longifolia* (Horsemint)	*Yersinia ruckeri*	*Oncorhynchus mykiss*	0.0, 0.1, 0.2, and 0.3% of horsemint extract in 12 aquaria; 4 weeks	Oral—through feed	Significant rise in serum and blood immune indices	Heydari et al. (2020)
*Eichhornia crassipes* (water hyacinth)	*Vibrio harveyi*	*Channa punctata*	2.5% and 5% supplementary feed; 15 days	Oral—through feed	Increased disease resistance and fish immunity against the *Vibrio harveyi* infection	Verma et al. (2021)
*Coriandrum sativum*	*Yersinia ruckeri*	*Yersinia ruckeri*	0 (control), 0.5%, 1% and 2% of coriander seed extract (CSE); eight weeks	Oral—through feed	Promote growth, enhance immunity and resistance against *Yersinia ruckeri*	Naderi Farsani et al. (2019)

### Essential oils

Since the medieval period, essential oils (over 250 oils) have been used for several medicinal, cosmetic and pharmaceutical purpose (Baptista-Silva et al. 2020), (Wińska et al. 2019). Although they are most commonly used for their cosmetic purposes, in recent years, scientists and researchers around the world have been experimenting with their antimicrobial properties. The antibacterial properties of essential oils are now well recognised to be correlated with their chemical makeup, particularly the phenolic components. By interfering with and impairing the phospholipid bilayer of bacterial cell membranes, enzyme systems, and genetic material, essential oils also exhibit an antibacterial effect (Abdollahzadeh et al. 2014). These essential oils inhibit the production of toxic bacterial metabolites and sometimes even their growth. This usually occurs due to the interaction between the essential oils and the cytoplasm and/or the bacterial membrane which in turn affects their quorum sensing systems, i.e., bacterial pheromones (Anastasiou et al. 2019). Essential oils can be obtained from various parts of a plant such as twigs, bark, wood, roots, fruits, flowers, herbs, leaves, buds, and buds. Some examples of essential oils are fennel (*Foeniculum vulgare* Miller), cypress (*Cupressus sempervirens* L.), thyme (*Thymus vulgaris* L.), herb-of-the-cross (*Verbena officinalis* L.) and pine (*Pinus sylvestris*) (Gómez-Estaca et al. 2010). Table 5 is a summary of the different essential oils along with their source and effectiveness against different bacterial strains that afflict marine fish.

**Table 5:** A summary of studies highlighting various essential oils against different bacterial fish pathogens and their antimicrobial activity range

Essential oil	Source	Bacterial strain	Antimicrobial activity	References
Clove oil (*Syzygium aromaticum*)	Clove flower bud	*Proteus mirabilis*	+ +	Wińska et al. (2019)
		*Staphylococcus aureu*	+	
Eucalyptus oil	Fresh leaves and branch tops of the eucalyptus plant	*Staphylococcus aureus* *Streptococcus iniae* *Vibrio harveyi*	+ +	Wińska et al. (2019)
Lavender oil, also known as *Lavandula angustifolia*	Flower spikes of certain species of lavender	*Staphylococcus aureus* *Clostridium perfringens*	+ + +	Wińska et al. (2019)
		*Shigella sonnei*	+ +	
		*Listeria monocytogenes*	+	
Rosemary essential oil	Derived from the aromatic herb *Rosmarinus Officinalis*	*Shigella sonnei* *Yersinia enterocolitica* *V. anguillarum*	+ + +	Gómez-Estaca et al. (2010) ; Anastasiou et al. (2019)
		*Vibrio parahaemolyticus* *E. anguillarum*	+ +	
		*Shewanella putrefaciens*	+	
Orange oil	Cells within the rind of an orange fruit	*V. anguillarum*	+ +	Mancuso et al. (2019)
		*Photobacterium d. P* *S. aureus 790*	+	

Legend: +  +  +  = highly effective; +  +  = effective; +  = minimally effective

In a study including 14 different essential oils against different fish pathogens, it was observed that most essential oils exhibited antimicrobial properties. *Aeromonas* spp. was susceptible to all antimicrobials that were used in that study. *Cinnamommum camphora* exhibited the most activity against most of the isolates with strong antimicrobial activity against Gram-positive and Gram-negative bacteria (Klūga et al. 2021). Several studies conducted in this field show that Gram-positive bacteria fish pathogens are more susceptible to essential oils than Gram-negative bacteria (Wu et al. 2014). Most of the research studies done in this field is conducted under the food preservation and foodborne pathogens. Research toward using essential oils as an antimicrobials for pathogens in the pisciculture industry is upcoming and ongoing.

## Conclusion and future prospects

Bacterial diseases in the pisciculture industry are commonplace. While efficient mitigation methods exist, it has become increasingly easy to understand that they possess harmful, severe effects and are far from sustainable. Emerging dangers, such as antibiotic-resistant strains, biomagnification, and water pollution, have become a reality. Vaccines are uneconomical, may be labour-intensive, and have not been developed for several fish pathogens. Thus, while they function as an effective prevention option, there continues to be a deficit.

As discussed, antimicrobial peptide administration is an emerging alternative, and some preliminary studies show promise. However, it requires efficacy improvements, better delivery mechanisms and other features necessary for widespread commercial application of the treatment method. The use of probiotics, medicinal plants, and essential oils to treat diseases in fish has also seen a rise in recent years. Their immunomodulatory properties could prove highly advantageous to prevent and cure various fish bacterial illnesses in the coming years.

It is imperative to note that the applicability of each method is subjective and may depend on the fish species, the causative pathogen, the stage of development of the disease, the state of the cultivation set-up, and several other factors making it rather difficult to draw concrete lines over which method of treatment may triumph. Notwithstanding, it is well established that in the forthcoming decade, it is of vital importance to move towards better and more sustainable practices while dealing with bacterial fish diseases in the mariculture industry to ensure a safer environment for consumers and producers and food security for an ever-increasing population.

## Data Availability

The data will be made available on request.
